# Place of Serology in the Diagnosis of Zoonotic Leishmaniases With a Focus on Visceral Leishmaniasis Due to *Leishmania infantum*

**DOI:** 10.3389/fcimb.2020.00067

**Published:** 2020-02-25

**Authors:** Maude F. Lévêque, Laurence Lachaud, Loïc Simon, Emilie Battery, Pierre Marty, Christelle Pomares

**Affiliations:** ^1^Laboratoire de Parasitologie-Mycologie, Centre Hospitalier Universitaire et Université de Montpellier, UMR MIVEGEC, Centre National de Référence des Leishmanioses, Montpellier, France; ^2^Laboratoire de Parasitologie-Mycologie, Centre Hospitalier Universitaire l'Archet, INSERM, U1065, C3M, Virulence microbienne et signalisation inflammatoire - Université de la Côte d'Azur, Faculté de Médecine, Laboratoire associé au Centre National de Référence des Leishmanioses, Nice, France

**Keywords:** visceral leishmaniasis, tegumentary leishmaniasis, zoonosis, serology, diagnosis

## Abstract

Leishmaniases are a group of parasitic diseases transmitted through the bite of female phlebotomine sandflies. Depending on the *Leishmania* species, the reservoirs can be humans (anthroponosis) or different animals (zoonosis). Zoonotic leishmaniasis present several clinical forms in function of the species involved: visceral leishmaniasis (VL), cutaneous leishmaniasis (CL), and muco-cutaneous leishmaniasis (MCL). The biological diagnosis is of utmost importance because the clinical features are not specific. In addition to parasitological and molecular biology (polymerase chain reaction, PCR) assays, serology is routinely used for the diagnosis of leishmaniasis. Indeed, although PCR is more sensitive than serological assays, its implementation is limited to referral laboratories and research centers. Therefore, serology is still a key element for their diagnosis. Here, we discuss the different serological assays available for the diagnosis of zoonotic leishmaniasis. We will review the enzyme-linked immunosorbent assay, immunofluorescence antibody test, immunochromatography test (ICT), direct agglutination test, and western blot as well as the different diagnostic strategies in function of the clinical form (VL, CL, and MCL). We will also discuss the place of serology for detecting asymptomatic carriers and for the follow-up of VL. Depending on the laboratory, different assays can be used, from ICT, which is appropriate for field testing, to a combination of serological tests to improve the sensitivity.

## Introduction

Leishmaniases are diseases caused by *Leishmania* parasites transmitted through the bite of female phlebotomine sandflies. The disease spectrum ranges from a visceral form to localized skin lesions and, occasionally mucosal lesions. The clinical manifestations depend in part on the involved *Leishmania* species. More than 20 species have been identified, and many of them cause zoonoses (Akhoundi et al., 2016). Humans become accidentally infected when they enter an endemic area. *Leishmania major, Leishmania guyanensis, Leishmania braziliensis*, and *Leishmania infantum* are the main zoonotic species detected in infected humans. *L. major* is responsible of Old World cutaneous leishmaniasis (CL) that occurs preferentially in rural desert areas where rodents are endemic. *L. guyanensis* and *L. braziliensis* are agents of CL and mucosal leishmaniasis in the New World, where the reservoirs are forest animals. *L. infantum* is essentially responsible of a domestic zoonosis that involves dogs and is present in foci in the Mediterranean region and in South America. In dogs, the disease presents a generalized cutaneous and an ovarian form that are spontaneously fatal. However, many cases are asymptomatic (Lachaud et al., 2002). It should be noted that the Iberian hare (*Lepus granatensis*) and a wild rabbit (*Oryctolagus cuniculus*) have recently been implicated as reservoirs of visceral leishmaniasis (VL) caused by *L. infantum* in Spain (Molina et al., 2012; Díaz-Sáez et al., 2014). In humans, *L. infantum* disease is mainly visceral (VL) (Burza et al., 2018). *L. infantum* is also responsible of rare forms, such as muco-cutaneous leishmaniasis (MCL) and the lymph node form that are considered close (or related) to VL and are similarly managed. CL due to *L. infantum* is infrequent and can go unnoticed and heal spontaneously.

Currently, several specific techniques are available for the diagnosis of VL and tegumentary leishmaniasis (TL: CL and MCL). The direct diagnosis is considered the gold standard for VL and TL, whatever the species involved. It is based on the direct microscopic examination of colored smears, culture, and PCR analysis of any available sample: whole blood, bone marrow, spleen biopsy, liver biopsy and lymph nodes (for VL), skin (for CL), and mucosal samples (for MCL). Molecular methods (PCR) display the best sensitivity and specificity, but their implementation requires specific material and well-equipped laboratories (de Ruiter et al., 2014). Direct parasite examination may be performed in any laboratory equipped with a light microscope but lacks sensitivity and require invasive procedures. Culturing parasites isolated from patients samples might improve the diagnostic sensitivity but is restricted to reference centers and is inappropriate for urgent diagnosis. Therefore, indirect diagnosis based on serology has been an alternative strategy for many decades, and still remains widely used due to its ease of implementation using serum or plasma samples. Various serological methods are available: immunofluorescence antibody test, enzyme-linked immunosorbent assay, immunochromatography test, direct agglutination test, western blot, and latex agglutination test (reviewed in Asfaram et al., 2018). Other assays are also under development with promising results for some of them (Maalej et al., 2003; Celeste et al., 2014; Portela et al., 2018).

In this review article we will critically describe the most frequently used serological assays for the diagnosis of VL due to *L. infantum*, and their interest for CL and MCL.

## Materials and Methods

We identified articles in English language in several databases, including PubMed, Science Direct, Google Scholar and Web of Science, using a combination of terms (serology, *Leishmania*, direct agglutination test, ELISA, western blot, diagnosis, immunofluorescence antibody test, immunochromatic test, visceral leishmaniasis, cutaneous leishmaniasis, muco-cutaneous leishmaniasis, and tegumentary leishmaniasis). The articles reviewed dated from 1987 to 2019. We identified other articles by reviewing the reference list of the retrieved articles. Only the studies performed on human specimens with demonstration of *Leishmania* parasites by microscopy, culture or PCR as confirmatory diagnostic method for VL were included. We excluded articles providing insufficient data information, those using a small sample size (*n* < 30) as well as the articles solely on *L. donovani* and *L. tropica*. For each method, we will present the most relevant assays, and discuss the interest of the different techniques in function of the clinical presentation and available laboratory equipment.

## Serological Diagnosis of Visceral Leishmaniasis Due to *L. infantum*

In humans, VL is characterized by irregular fever, weight loss, enlargement of spleen and liver, and anemia. However, VL may have a broad clinical spectrum, ranging from sub-clinical to the complete clinical form. As symptoms are not specific, VL diagnosis can be challenging. This is an issue because early diagnosis is required for rapid management. Indeed, if left untreated, VL is fatal in more than 95% of cases. In endemic areas, physicians are aware of this disease. On the other hand, in non-endemic areas, it is important that physicians know about this disease (Stensvold et al., 2019) to order the correct test when suspecting VL. Serological assays are widely used in leishmaniasis endemic areas. In this chapter, we will assess the technical performances of the different Enzyme-Linked Immunosorbent Assay (ELISA), Immunofluorescence Antibody Test (IFAT), Direct agglutination test (DAT), Immunochromatic tests (ICT) and Western blot (WB) assays. In the next chapters, we will evaluate their values for the follow-up of treated patients, in immunocompromised patients, and in asymptomatic carriers.

### Enzyme-Linked Immunosorbent Assay

**Figure 1:**
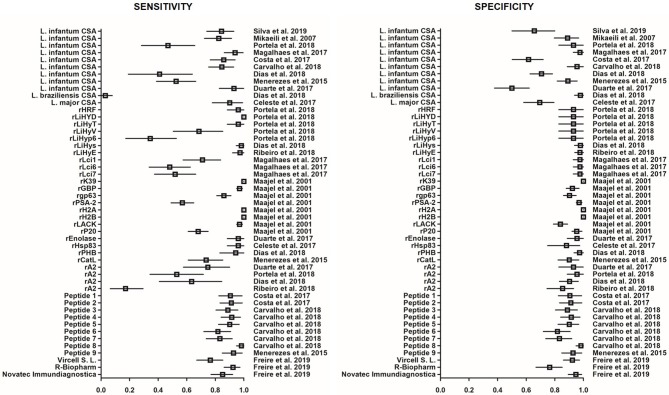
Forest plot showing the sensitivity and specificity of ELISA based on various antigens for the diagnosis of VL in immunocompetent patients. Each square represents the sensitivity or specificity estimated in the corresponding study. Horizontal lines represent the 95% Confidence Interval. CSA, crude soluble antigens; *r*, recombinant; Peptide 1, LSFPFPG; Peptide 2, CFTSFSPY; Peptide 3, SGAPRANNSGDASA; Peptide 4, GLSGEGSPASPEPRLAGGGGGADTQSTT; Peptide 5, DGKPKENQKTARES; Peptide 6, VADSGSASSEDGGSAKP; Peptide 7, PRKADPNDTTPQ; Peptide 8, GDSPPSDSPQNNQDRNRNQN; Peptide 9, QTSGSTTPGPTTTTK.

ELISA is a commonly used method for VL serological screenings. For this test, the well-bottoms are coated with the antigen(s) of interest, and then the reaction between the antibodies contained in the patient serum and the antigens is quantified using enzyme-labeled anti-human immunoglobulins. This is an old assay that has been widely used for diagnostic purposes since its first description (Engvall and Perlmann, 1972). For VL, several ELISA assays based on different antigens have been developed. Diagnostic commercial kits and home-made tests are available as well as methods used only for research with the aim of developing new tests to improve VL diagnosis. The sensitivity and specificity of the different tests are influenced by the type of antigen used. Crude soluble antigens (CSA), recombinant proteins and synthetic peptides have been assessed in different settings, yielding heterogeneous results (Figure 1).

Evaluation of the commercial assays *Leishmania* ELISA IgG + IgM (Vircell SL, Granada, Spain), Ridascreen *Leishmania* Ab (R-Biopharm AG, Germany) and NovaLisa *Leishmania infantum* IgG (Novatec Immundiagnostica GMBH ELISA Germany) showed that sensitivity and specificity range from 77.5 to 93.8% and from 77.2 to 96.2%, respectively, in patient populations without HIV (Freire et al., 2019; Figure 1). In HIV-positive patients, sensitivity and specificity drop and range from 63.2 to 78.9% and from 60 to 97.4%, respectively (Deniau et al., 2003; Freire et al., 2019). In most of these commercial assays, the used antigen is derived from cultures of *L. infantum* promastigotes.

In home-made assays, antigens are either CSAs, or the recombinant *Leishmania* K39 protein (rK39) (Deniau et al., 2003; Elmahallawy et al., 2014; Portela et al., 2018). CSA lysates are generally obtained by repeated freezing and thawing of live promastigotes grown in culture followed by cold centrifugation. The sensitivity and specificity of ELISA performed with *L. infantum* CSA range from 40 to 96.7% and from 63 to 100%, respectively (Duarte et al., 2017; Magalhães et al., 2017; Carvalho et al., 2018; Dias et al., 2018a; Portela et al., 2018). CSAs isolated from other *Leishmania* species (i.e., *L. major* or *L. braziliensis*) generally display lower performance (sensitivity between 1 and 87.5%; specificity from 21.3 to 100%) (Celeste et al., 2014; Menezes-Souza et al., 2015; Dias et al., 2018b; Ribeiro et al., 2018). This sensitivity variation could result from antigenic differences among endemic isolates and cultured parasites, while specificity may depend on cross-reactivity with other endemic pathogens.

Cross reactivity has been found for tuberculosis, leprosy, American and African trypanosomiasis, malaria, toxoplasmosis, auto-immune disease, sepsis, infective endocarditis, lymphoma, and hepatic insufficiency (Deniau et al., 2003; Elmahallawy et al., 2014; Freire et al., 2019). The performance of these tests is also influenced by the preparation quality, and the antigen purity and stability (immunogenic properties may decrease over time). On the other hand, assays based on the rK39 protein, which is also used for home-made ELISA, give more standardized results. On the basis of the information included in some of the retrieved articles, rK39 was mainly purchased from S.G. Reed, Corixa Inc. (Seattle, USA) (Deniau et al., 2003; Maalej et al., 2003; Lakhal et al., 2012) InBios International (Seattle, USA) (De Almeida Silva et al., 2006), and Rekom Biotech (Granada, Spain) (Magalhães et al., 2017). Although heterogeneous results have been reported using ELISA based on this recombinant antigen in patient populations with and without (i.e., immunocompetent) HIV (Deniau et al., 2003; Elmahallawy et al., 2014), a meta-analysis indicated that in patients without HIV, rK39-based ELISA shows better performance than CSA-based ELISA (Maia et al., 2012).

In research, *Leishmania* histone extracts, synthetic peptides and various recombinant proteins that are conserved in different *Leishmania* species but show low similarity with other parasite or hosts proteins have been evaluated. ELISA using *Leishmania* histone extracts displays higher sensitivity (97.6%) and specificity (100%) than *L. infantum* CSA-based ELISA (Lakhal et al., 2012). Synthetic peptides and recombinant proteins offer the possibility of more standardized and uniform production of antigens that can improve test reproducibility. Newly tested recombinant antigens with sensitivity and specificity values higher than 95%, include histone H2A and H2B, gene B protein (rGBP), prohibin (rPHB), enolase, histamine-releasing factor (rHRF), hypothetical proteins (rLiHyD, rLiHyT, rLiHyS, rLiHyE), homolog receptor for activated C kinase (rLACK), and heat shock protein 83 (rHsp83) (Maalej et al., 2003; Celeste et al., 2014; Duarte et al., 2017; Dias et al., 2018a,b; Portela et al., 2018; Ribeiro et al., 2018). Of note, rHRF, rLiHyD, and rLiHyT might be suitable for the serological monitoring of patients with VL after treatment (Portela et al., 2018). Synthesized peptides are potentially more stable, cheaper and easier to produce than recombinant proteins. Some peptides performed well for the diagnosis of VL and displayed little cross-reactivity with sera from patients infected by other parasitic pathogens (Menezes-Souza et al., 2015; Costa et al., 2017; Carvalho et al., 2018). This large panel of molecules, used either alone or in combination, may offer promising alternatives to improve the serodiagnosis of VL by ELISA. However, additional large-scale studies are needed to standardize the experimental conditions for accurate comparisons in different settings.

### Immunofluorescence Antibody Test

IFAT slides are prepared by fixing *Leishmania* promastigotes on glass slides. Then, the serum of interest is serially diluted to obtain quantitative results. Revelation is done using an anti-human immunoglobulin labeled with a fluorescent dye as secondary antibody. The results correspond to a titer (i.e., the last serum dilution yielding a fluorescence signal) evaluated by microscopic examination. Therefore, to perform this assay, the laboratory must have a fluorescent microscope and personnel trained in slide reading (Millesimo et al., 1996). IFAT has been widely used for the diagnosis of VL since the 1970's. Commercial assays and home-made methods are available. In home-made methods, *L. infantum* is the most frequently used species, whereas the use of *Leishmania amazonensis* is rare (Silva et al., 2005; De Almeida Silva et al., 2006; Mikaeili et al., 2007; Pedras et al., 2008). Studies are available for the following IFAT commercial assays: IFI Leishmaniose Humana (Fundação Oswaldo Cruz), *Leishmania* IFA IgG (Vircell), IFI—Leishmaniose Humana (Bio-Manguinhos), and Leishmania-Spot IF (bioMérieux) (Peruhype-Magalhães et al., 2012; Cota et al., 2013; Ntais et al., 2013; Varani et al., 2017; Krepis et al., 2018; Freire et al., 2019). Freire et al. compared IFI—Leishmaniose Humana and *Leishmania* IFA IgG and found that the latter displayed higher specificity in patients without HIV infection (Freire et al., 2019). As observed for the ELISA tests, IFAT sensitivity is lower in HIV-positive patients than in non-immunocompromised patients (Bangert et al., 2018; Freire et al., 2019). Sensitivity and specificity for VL diagnosis range from 78.8 to 100% and from 82.3 to 96.2%, respectively, in HIV-negative patients (Krepis et al., 2018; Freire et al., 2019), and from 51 to 78.4% and from 79 to 99.2%, respectively, in HIV-positive patients (Cota et al., 2012; Bangert et al., 2018). Cross-reactivity with Chagas disease, tuberculosis, toxoplasmosis, malaria, typhoid fever, or brucellosis may lead to false-positive results (Sarkari et al., 2014; Sakkas et al., 2016; Bangert et al., 2018). Due to the many protocols used for IFAT and the absence of international standards, it is difficult to compare data from different studies. Indeed, the positivity threshold may vary (1/40 in some studies, 1/80 in others) in function of the studied population and the assay set-up (Maia et al., 2012; Bangert et al., 2018). Finally, IFAT has been used not only for the diagnosis of VL, but also for the detection of asymptomatic carriers of CL and MCL (Sakru et al., 2007; Sarkari et al., 2014).

### Direct Agglutination Test

DATs have been used for the diagnosis of VL for more than 30 years and are still widely implemented in South America, Iran and to a lesser extent in Europe (de Korte et al., 1990; Nigro et al., 1996; Mikaeili et al., 2007; Farajnia et al., 2008; Farahmand and Nahrevanian, 2016; Bangert et al., 2018; Sarkari et al., 2018; Kühne and Büscher, 2019). The principle of this assay is to test whether agglutination occurs when serial dilutions of patient serum are mixed with stained killed *Leishmania* sp. promastigotes. This is a semi-quantitative assay, and the results correspond to a titer (Chappuis et al., 2006; Freire et al., 2019). The assay can be set up using *Leishmania donovani* provided by KIT Biomedical Research (Amsterdam, The Netherlands) (Pedras et al., 2008; Bangert et al., 2018) and home-made processed, or freeze-dried *L. infantum* promastigotes available from the Prince Leopold Institute (Antwerp, Belgium) and The Royal Tropical Institute (Amsterdam, The Netherlands) (Pedras et al., 2008; Oliveira et al., 2011). DAT results seem not to be influenced by the used species (*L. donovani* or *L. infantum*) (Pedras et al., 2008). The assay preparation requires culturing live parasites as antigens, and difficulties in the standardization of this step may lead to variation in the test results (Maia et al., 2012). Moreover, 2-mercaptoethanol (2-ME) is needed for the test. As this compound has health and environmental impacts, a new assay has been set up with N-acetylcysteine instead of 2-ME as sample diluent to improve its biosafety (Oliveira et al., 2011). In addition, a new DAT protocol (DAT-LPC) has been published with shorter incubation time (4 h compared with 18 h of the DAT KIT from the Royal Tropical Institute of Amsterdam) (Oliveira et al., 2009, 2017; Ntais et al., 2013). The last step is the reading of the end-titer. The end-titer depends on the used protocol, and varies from ≥1/100 for DAT-LPC, 1/1,600 for the DAT from KIT-Biomedical Research, to 1/3,200 (Pedras et al., 2008; Adams et al., 2012; Oliveira et al., 2013; Bangert et al., 2018; Kühne and Büscher, 2019). However, these variations do not seem to influence the test sensitivity and specificity for the clinical diagnosis of VL (Chappuis et al., 2006). In Brazil, a multicenter study reported good inter-laboratory reproducibility, but highlighted some inter-operators' variations concerning the end-titer reading, especially for low titers (Adams et al., 2012; Oliveira et al., 2017). DAT result interpretation could be standardized by using photographs of training plates validated by reference laboratories (Adams et al., 2012; Oliveira et al., 2017). As this assay can be performed with limited equipment, it is suitable for first-line laboratories (Pedras et al., 2008; World Health Organization, 2010a; Adams et al., 2012). In addition, this assay has good sensitivity and specificity in non-immunocompromised patients (sensitivity: 70.5–99% and specificity: 89.2–100%) and also in HIV-positive patients (sensitivity: 89.1–91.3% and specificity: 89.3–89.7%) (Mikaeili et al., 2007; Cota et al., 2013; Oliveira et al., 2013; Bangert et al., 2018; Freire et al., 2019). False-positive results were observed in patients infected by malaria or other parasites, such as trypanosomatids (Maia et al., 2012; Bangert et al., 2018). Thus, this assay can be used for the diagnosis of VL and seroprevalence studies with acceptable costs and good sensitivity and specificity in populations with and without HIV (Bangert et al., 2018).

### Immunochromatic Tests

Antigen-based ICTs are the most adapted for field testing (World Health Organization, 2010a). These assays are easy to perform, quick and user-friendly, thus allowing their use in remote areas where access to health facilities can be challenging (World Health Organization, 2010a; Elmahallawy et al., 2014). The test consists in a nitrocellulose membrane pre-coated with the rK39 antigen (few tests were developed with rKE16) (World Health Organization, 2010b). A drop of finger-prick blood or of whole blood, plasma or serum from venipuncture can be used. The results are qualitative, and the assay is considered positive when two bands are observed: the control band and the positive test band. Since the end of the 1990's, several brands and versions of the assay have been commercialized and used in endemic areas (Chappuis et al., 2006). WHO evaluated some of the commercially available tests: DiaMed-IT LEISH (Bio-Rad Laboratories), Crystal®KA (Span Diagnostics Ltd.), Signal®-KAb (Span Diagnostics Ltd.), Kalazar Detect™ (InBios International Inc.), and Onsite Leishmania Ab Rapid Test (CTK Biotech Inc.). Other tests are also on the market: IT LEISH (Bio-Rad Laboratories), rK39-ICT (SD BIOLINE Leishmania Ab, Standard Diagnostics, Inc., South Korea), and Rapydtest (Diagnostic International Distribution S.p.A, Milan, Italy) (Brandonisio et al., 2002; Carvalho et al., 2003; World Health Organization, 2010b; Cota et al., 2013; Varani et al., 2017; Bangert et al., 2018; Assis et al., 2019; Freire et al., 2019). ICTs have been tested mainly for the diagnosis of anthroponotic leishmaniasis and less frequently for zoonotic leishmaniasis (Boelaert et al., 2014). Overall, ICT diagnostic performance was excellent in the Indian sub-continent (Sensitivity ranged from 92.8 to 100%) compared with East Africa and Brazil (Sensitivity ranged from 36.8 to 92%) where results were less consistent (World Health Organization, 2010b). Regarding the WHO results for the zoonotic VL due to *L. infantum* in Brazil, the best sensitivity was obtained for DiaMed-IT LEISH (Se: 92%) whereas the best specificity was for Signal®-KAb (Sp: 98.8%) (World Health Organization, 2010b). A study in Brazil assessed cost-effective strategies, and found that VL diagnosis by ICT followed by treatment with amphotericin B (liposomal formulation) is less expensive with high effectiveness (Assis et al., 2019). During outbreaks, this assay is very convenient at the point of care facilities for rapid identification of cases (Bangert et al., 2018). When evaluated for zoonotic VL, this assay has a good specificity, up to 100% in some studies; however, a negative result does not rule out the diagnosis of VL because sensitivity can be lower than 60%, especially in HIV-infected patients (Carvalho et al., 2003; Deniau et al., 2003; Cota et al., 2013; Freire et al., 2019). Therefore, some authors recommend not to use ICT as a screening test for VL, especially in HIV-positive patients (Cota et al., 2013; Varani et al., 2017). Performances may also vary according the patient age, with lower accuracy in children younger than 3 years (Cruz et al., 2006; Freire et al., 2019). Cross-reactivity with malaria, enteric fever, disseminated tuberculosis, sepsis, infective endocarditis, lymphoma, hepatic insufficiency, and *Toxoplasma gondii* has been reported (Farahmand and Nahrevanian, 2016; Freire et al., 2019). Finally, users should be aware that ICT can be positive in asymptomatic carriers and for long time after treatment. Therefore, these assays cannot discriminate between VL relapse and other pathologies, like all the other serological assays (Elmahallawy et al., 2014).

### Western Blot

By the end of the 1980's, WB assays with potential diagnostic value had been developed for the diagnosis of human VL (dos Santos et al., 1987; Evans et al., 1989). In 1992, Mary et al. developed and evaluated a new WB test in 137 patients in the south of France. They demonstrated that the 14 and 16 kDa antigens were the most specific, with a sensitivity and specificity of 100 and 98%, respectively (Mary et al., 1992). The test was then marketed (LEISHMANIA WB IgG, LDBIO Diagnostics, France), and is currently used as a VL confirmation technique in France (Haute Autorité de Santé, 2017). Other studies in patients with VL due to *L. infantum* also highlighted the excellent results of this test that targets different antigenic fractions (Tebourski et al., 1994; Marty et al., 1995; Santos-Gomes et al., 2000; Pinedo-Cancino et al., 2013; Seyyedtabaei et al., 2017; Heidari et al., 2019). In the immunocompetent patients included in these studies, sensitivity (90–100%) and specificity (98–100%) were excellent. Nevertheless, the heterogeneity of antigen preparations as well as the choice of reference strain and specific bands do not allows comparing the results of the different studies. Moreover, the number of patients included in studies to evaluate these techniques was often too small (<30 patients) to obtain reliable statistical data. This technique has been evaluated also in immunocompromised patients, particularly patients with HIV, and for the detection of asymptomatic carriers in epidemiological studies (see below). However, this method is too complex to be used for field surveys, or if the daily demand is too important. Indeed, it is time-consuming, not automated, and quite expensive. Currently, it seems important to define which antigenic fractions are most relevant for this test and to assess their value for the diagnosis of VL and possibly for the therapeutic follow-up.

## Diagnosis of VL in Immunocompromised Patients

In immunocompromised patients, antibody-based tests often fail to diagnose VL. Thus, the choice of technique and the knowledge of its limits are essential. In these patients, the sensitivity of IFAT, ICT, DAT and ELISA is generally low (from 22 to 89%) (Deniau et al., 2003; Cota et al., 2012, 2013; Bangert et al., 2018; Freire et al., 2019). Probably the most robust test in this context is WB (75 to 91% of sensitivity) (Cota et al., 2012). Mary et al. found the same WB profile in *Leishmania-*infected patients with HIV and in immunocompetent patients, but for the 14-kD band (not or rarely recognized). Only 36% of these co-infected patients had positive results by IFAT and ELISA (Mary et al., 1992). Deniau et al. reported that 74% of patients co-infected by HIV and *Leishmania* have a “complete” WB profile (18-, 21-, 23-, and 31-kD bands), the profile described by Marty et al. as a good marker of symptomatic VL due to *L. infantum* (Marty et al., 1995; Deniau et al., 2003). Because of the lack of studies with DAT and WB comparison, it is difficult to perform recommendation regarding the performances of these assays in immunocompromised patients. Freire et al. found that the DAT-LPC has the best sensitivity (89.5%) whereas the best specificity was found for the *Leishmania* ELISA IgG + IgM (Vircell S. L.) (97.4%) (Freire et al., 2019). In immunocompromised patients, it is thus recommended to perform at least two serological assays in addition to the PCR.

## Asymptomatic Carriers

In endemic areas of *L. infantum*, asymptomatic *Leishmania* infection in human is usually ascertained by a positive Leishmanin skin test, PCR, or serological test in individuals who are otherwise in a healthy condition (Singh et al., 2014). The Leishmanin skin test remains remarkable for assessing endemicity levels, but its implementation is hardly feasible currently (Bekele et al., 2018). PCR is an alternative giving good results (Riera et al., 2008). Regarding serology, studies were conducted using WB to detect asymptomatic patients whose frequency varies depending on the context (Mary et al., 1992; Deniau et al., 2003; Sakru et al., 2007; Riera et al., 2008; Biglino et al., 2010; Saghrouni et al., 2012). For instance, 3.1% of blood donors are carriers of anti-*Leishmania* antibodies (Riera et al., 2008), and 54% of family members of patients with VL in Tunisia are positive (Riera et al., 2008; Saghrouni et al., 2012). In a larger study (526 healthy adults) in Italy, 7.41% had a positive WB test (Biglino et al., 2010). As dogs are the main reservoir of *L. infantum*, the studies on asymptomatic carriers using other techniques (ELISA, DAT…) were rarely carried out in humans but rather in dogs.

## Follow-up of Treated Patients

After VL diagnosis and treatment initiation, a clinical and biological follow-up should be performed to monitor the response to treatment. Biological parameters and frequency for the follow-up should be defined according to the laboratory facilities and the request of the physicians. Clinical follow-up consists of monitoring the vital signs and the decrease of the swelling of the liver and spleen. Serological follow-up (any serological test) is not recommended because antibodies may persist for life after an episode of VL. For instance, Carvalho et al. found that in up to 46% of patients, antibodies can be detected with an ICT assay for months after VL is cured (Carvalho et al., 2003). Therefore, qualitative assays, such as WB and ICT that do not give titers, have limited use for patient follow-up. In addition, WB high sensitivity is suitable for the screening of asymptomatic carriers, but not for VL follow-up. Similarly, ICT can give positive results in asymptomatic patients, and this makes the diagnosis of relapse or re-infection challenging, especially in endemic areas (Brandonisio et al., 2002; Carvalho et al., 2003; Maia et al., 2012; Elmahallawy et al., 2014). Quantitative serological assays (ELISA, IFAT, and DAT) also are not recommended for patient follow-up because of the slow decrease of the antibody titer after VL treatment (De Almeida Silva et al., 2006; Ferrua et al., 2009). It should be noted that the slow antibody reduction is not a sign of bad prognosis or poor therapeutic response (De Almeida Silva et al., 2006).

*Leishmania* DNA detection by quantitative PCR (qPCR) appears to be the reference method for monitoring the response to treatment and for detecting VL relapse or re-infection. The parasite DNA level measured by qPCR should progressively decrease and become undetectable after treatment initiation. The major drawbacks of this assay are the need of trained personnel, and of equipped facilities with separate rooms for DNA extraction, preparation of the reaction mix, and PCR automat to avoid contamination. The use of qPCR is becoming more widespread, allowing optimal patient management for both diagnosis and therapeutic monitoring by a reference laboratory.

## Comparison of Assays

Comparison of studies on the different serological assays highlights a huge variability in the conclusions. Indeed, the assay performances can changes depending on the population (children, HIV-positive patients, immunocompetent patients) (Deniau et al., 2003; Bangert et al., 2018; Krepis et al., 2018; Freire et al., 2019). Few studies compared WB with the other serological assays because WB is mainly used for epidemiological studies to detect asymptomatic carriers and less frequently in HIV-infected patients and for CL or MCL diagnosis (Carvalho et al., 2003; Deniau et al., 2003; Cota et al., 2012; Maia et al., 2012; Pomares et al., 2012; Krepis et al., 2018). For the other assays (DAT, ELISA, IFAT, and ICT), in addition to the specific limitation of each assay, HIV infection can influence the performance of some tests (ELISA, IFAT) more than others (western blot) (Deniau et al., 2003). Moreover, the impact of the geographic region on the test diagnostic accuracy cannot be excluded, as highlighted by the WHO evaluation of ICT (World Health Organization, 2010b). From our personal experience, while testing, in a population of patients from the Mediterranean basin, identical serological assays from those tested by Freire et al. (2019) to diagnose VL in Brazil, different sensitivity and specificity were found (manuscript under review). One of the explanations of these discrepancies could be the differences in antibody concentrations among the different geographic regions (World Health Organization, 2010b) or genetic diversity between *L. infantum* strains from different area. Thus, before implementing a diagnosis strategy, the assays available should be tested in the population that is concerned by the disease and according to the laboratory facilities. The type of control population and the chosen threshold also may influence the assay specificity (Chappuis et al., 2006). All these considerations explain why there is no consensus on the best serological assay for the diagnosis of zoonotic VL. Most studies agree that rK39-based ICT represents a good solution in terms of user-friendliness, field applicability, and performance (Delgado et al., 2001; Carvalho et al., 2003; Deniau et al., 2003; de Assis et al., 2011; Maia et al., 2012; Peruhype-Magalhães et al., 2012; Cota et al., 2013; Boelaert et al., 2014; Bangert et al., 2018; Krepis et al., 2018; Freire et al., 2019). However, this assay should always be associated with at least another serological assay to increase the diagnosis sensitivity. Several studies suggested the use of DAT in addition to ICT because its gives reliable results in patients with HIV (Cota et al., 2012, 2013; Bangert et al., 2018; Freire et al., 2019). In conclusion, when choosing a serological test, its feasibility, sustainability, local population, and field applicability should be taken into account (Elmahallawy et al., 2014). When considering the cost of VL diagnosis and treatment, the most cost-effective strategy appears to be diagnosis using ICT followed by treatment with liposomal amphotericin B (Assis et al., 2019).

## Serological Diagnosis of Human Tegumentary Leishmaniasis Due to Zoonotic Species

CL affect between 1 and 1.5 million people per year worldwide (den Boer et al., 2011). Generally, cutaneous lesions are crusted papules or ulcers on exposed skin, localized at the site of the parasite inoculation by sandflies. In the Old World, the most consistently zoonotic dermotropic species are *L. major* (Central Asia, West, North and East Africa, Near and Middle East). In the New World, they include species with a large South American distribution (*L. amazonensis, L. braziliensis*, and *L*. *guyanensis*), species more localized in Central America (*L. mexicana, L. panamensis*), and other species with a restricted geographical territory (*L. peruviana, L. naiffi*, and the two Brazilian species *L. shawi* and *L. lainsoni*). MCL is a specific clinical entity mainly due to *L. braziliensis* that is widespread from southern Mexico to northern Argentina. The metastatic mucosal involvement is secondary to the cutaneous lesion and appears several years after the first skin lesions. In several studies, serological tests have been used for CL and MCL diagnosis because they are minimally invasive and easy to perform. However, their lack of sensitivity and specificity greatly limits their diagnostic usefulness. In TL, molecular and parasitological (microscopic examination, culture) diagnosis remain the reference standards (World Health Organization, 2010a). Molecular biology either directly from the sample or from cultivated parasite allows species identification required to adapt the treatment. For TL caused by *L. infantum*, case reports and literature data show that WB could contribute to the biological diagnosis (del Giudice et al., 1998). Similarly, for CL due to *L. major*, 47 and 100% of infected patients were identified as positive by IFAT and WB, respectively, in Algeria, Morocco, and Tunisia (Pomares et al., 2012), and 96.7% by WB in Iran (Ashrafmansouri et al., 2015). ICT and rK39 ELISA assays gave positive results only in 10.2 and 28.8%, respectively, of soldiers infected in Iraq or Afghanistan (Hartzell et al., 2008). In the American continent, initial studies on TL/CL in Brazil reported low sensitivities for IFAT (27.7%) and ELISA (66.9%) (Guimarães et al., 1990). In another study, their sensitivity was 75.4 and 95.7%, respectively (Barroso-Freitas et al., 2009). A new assay based on the recombinant rLb6H antigen displayed 100.0% sensitivity and 98.5% specificity in sera from patients with American TL (Sato et al., 2017). The same authors also found that ELISA assays based on novel antigens show better sensitivity and specificity for the diagnosis of American TL compared with the standard ELISA based on soluble *Leishmania* antigens (Zanetti et al., 2019). In conclusion, the serological assay for the routine diagnosis of TL remains to be defined. Additional well-conducted studies are needed to clearly define the interest of serology for TL diagnosis. Currently, WB should be preferred when skin sampling is not easy to perform or in the case of MCL. However, in order to confirm the diagnosis of TL, parasitological (microscopic examination, culture) and molecular diagnosis should be performed (World Health Organization, 2010a).

## Concluding Remarks

VL, CL, and MCL are the clinical expression of zoonotic leishmaniases. VL is a life-threatening disease and accurate diagnosis is required for its prompt and correct management. False-negative test results may lead to a fatal outcome if the patient is left untreated. On the other hand, false-positive test results could prompt physicians to treat the patient with unnecessary toxic molecules (Chappuis et al., 2006; Freire et al., 2019). This highlights the fact that the diagnosis of zoonotic leishmaniases is based not only on the result of a biological assay, but also on the patient's clinical examination, the understanding of the assay performance, and the follow-up and re-evaluation of the diagnostic hypotheses. The currently available serological assays have specific limits and advantages, and the choice of the assay(s) to be implemented in laboratories should be based on their performance, feasibility, and cost-effectiveness ratio. In remote areas, ICT is a good option for field testing, whereas in equipped laboratories, a panel of assays can be performed to increase sensitivity (Table 1). Among the different ELISA assays, performances vary, and this should be taken into account as well as the *in-situ* validation when choosing a test. IFAT is quite complicated to set up because of the need of trained personnel and a fluorescence microscope. Several studies promoted DAT; however, the lack of commercially available kits limits its diffusion. In immunocompromised patients without any clinical sign and history of VL, the positivity of the WB indicates an asymptomatic carriage. Such patients are at risk of VL due to their immunodepression. Serological tests allow diagnosis of VL with a very limited invasive procedure as it is performed from a sample of peripheral blood. In France, health policies recommend the use of PCR for the diagnosis of VL, CL, and MCL, and the follow-up of VL. Serological diagnosis should be performed first by ELISA or IFAT for the diagnosis of VL. Then, WB is used as a confirmatory assay when at least one of these screening assays is positive (Haute Autorité de Santé, 2017). Currently, PCR is necessary for the accurate diagnosis of VL if the serological test results are negative, especially in immunocompromised patients. Indeed, PCR is more sensitive than parasitological diagnosis made by microscopic examination and parasite culture. During the follow-up, PCR allows the accurate monitoring of the response to treatment. However, to date, this assay is limited to referral laboratories and research centers.

**Table 1:** Recommended assays according to the healthcare facility level.

**Healthcare facility level**	**Recommended assays**
Field application	ICT
Laboratory with minimal equipment	ICT plus DAT
Laboratory with intermediate equipment	2 serological assays among ELISA, DAT, ICT ± microscopy and culture, if microscope and trained personnel available
Reference laboratory	Serological assays (ELISA, DAT, ICT, western blotting, IFAT), microscopy examination, culture, and PCR

In conclusion, serology is a good tool for VL diagnosis and when possible, at least two assays should be performed to increase the sensitivity. Nevertheless, laboratory assays should always be considered with the clinical signs, and a diagnosis of TL should not be ruled out based only on a negative test result.

## Author Contributions

LL, ML, PM, and CP drafted the work, provide approval for publication of the content, contributed conception, and design of the study. LS revised it critically for important intellectual content. ML realized the figure. EB and LS wrote sections of the manuscript. All authors contributed to manuscript revision, read, and approved the submitted version.

### Conflict of Interest

The authors declare that the research was conducted in the absence of any commercial or financial relationships that could be construed as a potential conflict of interest.

## References

[B1] Adams E. R. Jacquet D. Schoone G. Gidwani K. Boelaert M. Cunningham J. (2012). *Leishmaniasis* direct agglutination test: using pictorials as training materials to reduce inter-reader variability and improve accuracy. PLoS Negl. Trop. Dis. 6:e1946. 10.1371/journal.pntd.0001946 23272257 PMC3521667

[B2] Akhoundi M. Kuhls K. Cannet A. Votýpka J. Marty P. Delaunay P.. (2016). A historical overview of the classification, evolution, and dispersion of *Leishmania* parasites and sandflies. PLoS Negl. Trop. Dis. 10:e0004349. 10.1371/journal.pntd.0004349 26937644 PMC4777430

[B3] Asfaram S. Hosseini Teshnizi S. Fakhar M. Banimostafavi E. S. Soosaraei M. (2018). Is urine a reliable clinical sample for the diagnosis of human visceral leishmaniasis? A systematic review and meta-analysis. Parasitol. Int. 67, 575–583. 10.1016/j.parint.2018.05.008 29775824

[B4] Ashrafmansouri M. Sarkari B. Hatam G. Habibi P. Abdolahi Khabisi S. (2015). Utility of western blot analysis for the diagnosis of cutaneous leishmaniasis. Iran J. Parasitol. 10, 599–604. 26811727 PMC4724837

[B5] Assis T. S. M. de Rabello A. Cota G. Werneck G. L. Azeredo-da-Silva A. L. F. (2019). Cost-effectiveness analysis of diagnostic-therapeutic strategies for visceral leishmaniasis in Brazil. Rev. Soc. Bras. Med. Trop. 52:e20180272. 10.1590/0037-8682-0272-2018 30994802

[B6] Bangert M. Flores-Chávez M. D. Llanes-Acevedo I. P. Arcones C. Chicharro C. García E.. (2018). Validation of rK39 immunochromatographic test and direct agglutination test for the diagnosis of mediterranean visceral leishmaniasis in Spain. PLoS Negl. Trop. Dis. 12:e0006277. 10.1371/journal.pntd.0006277 29494596 PMC5849364

[B7] Barroso-Freitas A. P. T. Passos S. R. L. Mouta-Confort E. Madeira M. F. Schubach A. O. Santos G. P. L.. (2009). Accuracy of an ELISA and indirect immunofluorescence for the laboratory diagnosis of American tegumentary leishmaniasis. Trans. R. Soc. Trop. Med. Hyg. 103, 383–389. 10.1016/j.trstmh.2008.12.019 19211118

[B8] Bekele F. Belay T. Zeynudin A. Hailu A. (2018). Visceral leishmaniasis in selected communities of Hamar and Banna-Tsamai districts in Lower Omo Valley, South West Ethiopia: sero-epidemological and leishmanin skin test surveys. PLoS ONE 13:e0197430. 10.1371/journal.pone.0197430 29795589 PMC5967802

[B9] Biglino A. Bolla C. Concialdi E. Trisciuoglio A. Romano A. Ferroglio E. (2010). Asymptomatic *Leishmania infantum* infection in an area of northwestern Italy (Piedmont region) where such infections are traditionally nonendemic. J. Clin. Microbiol. 48, 131–136. 10.1128/JCM.00416-09 19923480 PMC2812267

[B10] Boelaert M. Verdonck K. Menten J. Sunyoto T. van Griensven J. Chappuis F. (2014). Rapid tests for the diagnosis of visceral leishmaniasis in patients with suspected disease. Cochrane Database Syst. Rev. 20:CD009135 10.1002/14651858.CD009135.pub2 PMC4468926 24947503

[B11] Brandonisio O. Fumarola L. Maggi P. Cavaliere R. Spinelli R. Pastore G. (2002). Evaluation of a rapid immunochromatographic test for serodiagnosis of visceral leishmaniasis. Eur. J. Clin. Microbiol. Infect. Dis. 21, 461–464. 10.1007/s10096-002-0739-8 12111603

[B12] Burza S. Croft S. L. Boelaert M. (2018). Leishmaniasis. Lancet 392, 951–970. 10.1016/S0140-6736(18)31204-2 30126638

[B13] Carvalho A. M. R. S. Mendes T. A. de O. Coelho E. A. F. Duarte M. C. Menezes-Souza D. (2018). New antigens for the serological diagnosis of human visceral leishmaniasis identified by immunogenomic screening. PLoS ONE 13:e0209599. 10.1371/journal.pone.0209599 30571783 PMC6301785

[B14] Carvalho S. F. G. Lemos E. M. Corey R. Dietze R. (2003). Performance of recombinant K39 antigen in the diagnosis of Brazilian visceral leishmaniasis. Am. J. Trop. Med. Hyg. 68, 321–324. 10.4269/ajtmh.2003.68.321 12685638

[B15] Celeste B. J. Arroyo Sanchez M. C. Ramos-Sanchez E. M. Castro L. G. M. Lima Costa F. A. Goto H. (2014). Recombinant *Leishmania infantum* heat shock protein 83 for the serodiagnosis of cutaneous, mucosal, and visceral leishmaniases. Am. J. Trop. Med. Hyg. 90, 860–865. 10.4269/ajtmh.13-0623 24615136 PMC4015579

[B16] Chappuis F. Rijal S. Soto A. Menten J. Boelaert M. (2006). A meta-analysis of the diagnostic performance of the direct agglutination test and rK39 dipstick for visceral leishmaniasis. BMJ 333:723. 10.1136/bmj.38917.503056.7C 16882683 PMC1592383

[B17] Costa L. E. Salles B. C. S. Santos T. T. O. Ramos F. F. Lima M. P. Lima M. I. S.. (2017). Antigenicity of phage clones and their synthetic peptides for the serodiagnosis of canine and human visceral leishmaniasis. Microb. Pathog. 110, 14–22. 10.1016/j.micpath.2017.06.020 28629727

[B18] Cota G. F. de Sousa M. R. de Freitas Nogueira B. M. Gomes L. I. Oliveira E. Assis T. S. M.. (2013). Comparison of parasitological, serological, and molecular tests for visceral leishmaniasis in HIV-infected patients: a cross-sectional delayed-type study. Am. J. Trop. Med. Hyg. 89, 570–577. 10.4269/ajtmh.13-0239 23836568 PMC3771302

[B19] Cota G. F. de Sousa M. R. Demarqui F. N. Rabello A. (2012). The diagnostic accuracy of serologic and molecular methods for detecting visceral leishmaniasis in HIV infected patients: meta-analysis. PLoS Negl. Trop. Dis. 6:e1665. 10.1371/journal.pntd.0001665 22666514 PMC3362615

[B20] Cruz I. Chicharro C. Nieto J. Bailo B. Cañavate C. Figueras M.-C.. (2006). Comparison of new diagnostic tools for management of pediatric mediterranean visceral leishmaniasis. J. Clin. Microbiol. 44, 2343–2347. 10.1128/JCM.02297-05 16825347 PMC1489479

[B21] De Almeida Silva L. Romero H. D. Prata A. Costa R. T. Nascimento E. Carvalho S. F. G.. (2006). Immunologic tests in patients after clinical cure of visceral leishmaniasis. Am. J. Trop. Med. Hyg. 75, 739–743. 10.4269/ajtmh.2006.75.739 17038704

[B22] de Assis T. S. M. Braga A. S. da C. Pedras M. J. Oliveira E. Barral A.. (2011). Multi-centric prospective evaluation of rk39 rapid test and direct agglutination test for the diagnosis of visceral leishmaniasis in Brazil. Trans. R. Soc. Trop. Med. Hyg. 105, 81–85. 10.1016/j.trstmh.2010.09.004 20970152

[B23] de Korte P. M. Harith A. E. Dereure J. Huigen E. Faucherre V. van der Kaay H. J. (1990). Introduction of an improved direct agglutination test for the detection of *Leishmania infantum* infection in southern France. Parasitol. Res. 76, 526–530. 10.1007/BF00931059 2199962

[B24] de Ruiter C. M. van der Veer C. Leeflang M. M. G. Deborggraeve S. Lucas C. Adams E. R. (2014). Molecular tools for diagnosis of visceral leishmaniasis: systematic review and meta-analysis of diagnostic test accuracy. J. Clin. Microbiol. 52, 3147–3155. 10.1128/JCM.00372-14 24829226 PMC4313130

[B25] del Giudice P. Marty P. Lacour J. P. Perrin C. Pratlong F. Haas H.. (1998). Cutaneous leishmaniasis due to *Leishmania infantum*. case reports and literature review. Arch. Dermatol. 134, 193–198. 10.1001/archderm.134.2.193 9487211

[B26] Delgado O. Feliciangeli M. D. Coraspe V. Silva S. Perez A. Arias J. (2001). Value of a dipstick based on recombinant RK39 antigen for differential diagnosis of American visceral leishmaniasis from other sympatric endemic diseases in Venezuela. Parasite 8, 355–357. 10.1051/parasite/2001084355 11802273

[B27] den Boer M. Argaw D. Jannin J. Alvar J. (2011). Leishmaniasis impact and treatment access. Clin. Microbiol. Infect. 17, 1471–1477. 10.1111/j.1469-0691.2011.03635.x 21933305

[B28] Deniau M. Cañavate C. Faraut-Gambarelli F. Marty P. (2003). The biological diagnosis of leishmaniasis in HIV-infected patients. Ann. Trop. Med. Parasitol. 97 (Suppl. 1), 115–133. 10.1179/000349803225002598 14678639

[B29] Dias D. S. Ribeiro P. A. F. Martins V. T. Lage D. P. Ramos F. F. Dias A. L. T.. (2018a). Recombinant prohibitin protein of *Leishmania infantum* acts as a vaccine candidate and diagnostic marker against visceral leishmaniasis. Cell. Immunol. 323, 59–69. 10.1016/j.cellimm.2017.11.001 29128045

[B30] Dias D. S. Ribeiro P. A. F. Salles B. C. S. Santos T. T. O. Ramos F. F. Lage D. P.. (2018b). Serological diagnosis and prognostic of tegumentary and visceral leishmaniasis using a conserved *Leishmania* hypothetical protein. Parasitol. Int. 67, 344–350. 10.1016/j.parint.2018.02.001 29408435

[B31] Díaz-Sáez V. Merino-Espinosa G. Morales-Yuste M. Corpas-López V. Pratlong F. Morillas-Márquez F.. (2014). High rates of *Leishmania infantum* and *Trypanosoma nabiasi* infection in wild rabbits (*Oryctolagus cuniculus*) in sympatric and syntrophic conditions in an endemic canine leishmaniasis area: epidemiological consequences. Vet. Parasitol. 202, 119–127. 10.1016/j.vetpar.2014.03.029 24774436

[B32] dos Santos J. I. Morgado M. G. Galvão-Castro B. (1987). Human visceral leishmaniasis: analysis of the specificity of humoral immune response to polypeptides of *Leishmania* donovani chagasi. Am. J. Trop. Med. Hyg. 37, 263–270. 10.4269/ajtmh.1987.37.263 2444121

[B33] Duarte M. C. Lage D. P. Martins V. T. Costa L. E. Salles B. C. S. Carvalho A. M. R. S.. (2017). Performance of *Leishmania braziliensis* enolase protein for the serodiagnosis of canine and human visceral leishmaniosis. Vet. Parasitol. 238, 77–81. 10.1016/j.vetpar.2017.03.024 28385540

[B34] Elmahallawy E. K. Sampedro Martinez A. Rodriguez-Granger J. Hoyos-Mallecot Y. Agil A. Navarro Mari J. M.. (2014). Diagnosis of leishmaniasis. J. Infect. Dev. Ctries. 8, 961–972. 10.3855/jidc.4310 25116660

[B35] Engvall E. Perlmann P. (1972). Enzyme-linked immunosorbent assay, elisa. 3. quantitation of specific antibodies by enzyme-labeled anti-immunoglobulin in antigen-coated tubes. J. Immunol. 109, 129–135. 4113792

[B36] Evans T. G. Krug E. C. Wilson M. E. Vasconcelos A. W. de Alencar J. E. Pearson R. D. (1989). Evaluation of antibody responses in American visceral leishmaniasis by ELISA and immunoblot. Mem. Inst. Oswaldo Cruz 84, 157–166. 10.1590/S0074-02761989000200003 2635749

[B37] Farahmand M. Nahrevanian H. (2016). Application of recombinant proteins for serodiagnosis of visceral leishmaniasis in humans and dogs. Iran Biomed. J. 20, 128–134. 10.7508/ibj.2016.03.001 26883952 PMC4949976

[B38] Farajnia S. Darbani B. Babaei H. Alimohammadian M. H. Mahboudi F. Gavgani A. M. (2008). Development and evaluation of *Leishmania infantum* rK26 ELISA for serodiagnosis of visceral leishmaniasis in Iran. Parasitology 135, 1035–1041. 10.1017/S003118200800454X 18561868

[B39] Ferrua B. Rol N. Michel G. Marty P. (2009). Antigenemia in patients with mediterranean visceral leishmaniasis. J. Clin. Microbiol. 47, 3760–3762. 10.1128/JCM.00649-09 19759231 PMC2772584

[B40] Freire M. L. Machado de Assis T. Oliveira E. Moreira de Avelar D. Siqueira I. C. Barral A.. (2019). Performance of serological tests available in Brazil for the diagnosis of human visceral leishmaniasis. PLoS Negl. Trop. Dis. 13:e0007484. 10.1371/journal.pntd.0007484 31318856 PMC6638734

[B41] Guimarães M. C. Celeste B. J. Franco E. L. (1990). Diagnostic performance indices for immunofluorescent tests and enzyme immunoassays of leishmaniasis sera from northern and north-eastern Brazil. Bull. World Health Organ. 68, 39–43. 2189584 PMC2393007

[B42] Hartzell J. D. Aronson N. E. Weina P. J. Howard R. S. Yadava A. Wortmann G. W. (2008). Positive rK39 serologic assay results in US servicemen with cutaneous leishmaniasis. Am. J. Trop. Med. Hyg. 79, 843–846. 10.4269/ajtmh.2008.79.843 19052290

[B43] Haute Autorité de Santé (2017). Actualisation des Actes de Biologie Médicale Relatifs au Diagnostic de la Leishmaniose (Saint-Denis).

[B44] Heidari S. Gharechahi J. Mohebali M. Akhoundi B. Mirshahvaladi S. Azarian B.. (2019). Western Blot analysis of *Leishmania infantum* antigens in sera of patients with visceral leishmaniasis. Iran J. Parasitol. 14, 10–19. 10.18502/ijpa.v14i1.713 31123464 PMC6511593

[B45] Krepis P. Krepi A. Argyri I. Aggelis A. Soldatou A. Papaevangelou V.. (2018). Childhood visceral leishmaniasis: distinctive features and diagnosis of a re-emerging disease. an 11-year experience from a tertiary referral center in Athens, Greece. Pediatr. Infect. Dis. J. 37, 419–423. 10.1097/INF.0000000000001797 28938257

[B46] Kühne V. Büscher P. (2019). The unknown nature of the antigen in the direct agglutination test for visceral leishmaniasis hampers development of serodiagnostic tests. Am. J. Trop. Med. Hyg. 100, 246–255. 10.4269/ajtmh.18-0740 30560773 PMC6367635

[B47] Lachaud L. Chabbert E. Dubessay P. Dereure J. Lamothe J. Dedet J. P.. (2002). Value of two PCR methods for the diagnosis of canine visceral leishmaniasis and the detection of asymptomatic carriers. Parasitology 125, 197–207. 10.1017/S0031182002002081 12358417

[B48] Lakhal S. Mekki S. Ben-Abda I. Mousli M. Amri F. Aoun K.. (2012). Evaluation of an enzyme-linked immunosorbent assay based on crude *Leishmania* histone proteins for serodiagnosis of human infantile visceral leishmaniasis. Clin. Vaccine Immunol. 19, 1487–1491. 10.1128/CVI.00257-12 22815147 PMC3428402

[B49] Maalej I. A. Chenik M. Louzir H. Ben Salah A. Bahloul C. Amri F.. (2003). Comparative evaluation of ELISAs based on ten recombinant or purified *Leishmania* antigens for the serodiagnosis of mediterranean visceral leishmaniasis. Am. J. Trop. Med. Hyg. 68, 312–320. 10.4269/ajtmh.2003.68.312 12685637

[B50] Magalhães F. B. Castro Neto A. L. Nascimento M. B. Santos W. J. T. Medeiros Z. M. Lima Neto A. S.. (2017). Evaluation of a new set of recombinant antigens for the serological diagnosis of human and canine visceral leishmaniasis. PLoS ONE 12:e0184867. 10.1371/journal.pone.0184867 28957332 PMC5619722

[B51] Maia Z. Lírio M. Mistro S. Mendes C. M. C. Mehta S. R. Badaro R. (2012). Comparative study of rK39 *Leishmania* antigen for serodiagnosis of visceral leishmaniasis: systematic review with meta-analysis. PLOS Negl. Trop. Dis. 6:e1484. 10.1371/journal.pntd.0001484 22303488 PMC3269412

[B52] Marty P. Lelièvre A. Quaranta J. F. Suffia I. Eulalio M. Gari-Toussaint M.. (1995). Detection by western blot of four antigens characterizing acute clinical leishmaniasis due to *Leishmania infantum*. Trans. R. Soc. Trop. Med. Hyg. 89, 690–691. 10.1016/0035-9203(95)90447-6 8594698

[B53] Mary C. Lamouroux D. Dunan S. Quilici M. (1992). Western blot analysis of antibodies to *Leishmania infantum* antigens: potential of the 14-kD and 16-kD antigens for diagnosis and epidemiologic purposes. Am. J. Trop. Med. Hyg. 47, 764–771. 10.4269/ajtmh.1992.47.764 1281966

[B54] Menezes-Souza D. Mendes T. A. Gomes M. de. S. Bartholomeu D. C. Fujiwara R. T. (2015). Improving serodiagnosis of human and canine leishmaniasis with recombinant *Leishmania braziliensis* cathepsin l-like protein and a synthetic peptide containing its linear B-cell epitope. PLoS Negl. Trop. Dis. 9:e3426. 10.1371/journal.pntd.0003426 25569432 PMC4287388

[B55] Mikaeili F. Fakhar M. Sarkari B. Motazedian M. H. Hatam G. (2007). Comparison of serological methods (ELISA, DAT and IFA) for diagnosis of visceral leishmaniasis utilizing an endemic strain. Iran J. Immunol. 4, 116–121. 17652852 10.22034/iji.2007.17188

[B56] Millesimo M. Zucca M. Caramello P. Savoia D. (1996). Evaluation of the immune response in visceral leishmaniasis. Diagn. Microbiol. Infect. Dis. 26, 7–11. 10.1016/S0732-8893(96)00168-X 8950522

[B57] Molina R. Jiménez M. I. Cruz I. Iriso A. Martín-Martín I. Sevillano O.. (2012). The hare (*Lepus granatensis*) as potential sylvatic reservoir of *Leishmania infantum* in Spain. Vet. Parasitol. 190, 268–271. 10.1016/j.vetpar.2012.05.006 22677135

[B58] Nigro L. Vinci C. Romano F. Russo R. (1996). Comparison of the indirect immunofluorescent antibody test and the direct agglutination test for serodiagnosis of visceral leishmaniasis in HIV-infected subjects. Eur. J. Clin. Microbiol. Infect. Dis. 15, 832–835. 10.1007/BF01701531 8950566

[B59] Ntais P. Sifaki-Pistola D. Christodoulou V. Messaritakis I. Pratlong F. Poupalos G.. (2013). Leishmaniases in Greece. Am. J. Trop. Med. Hyg. 89, 906–915. 10.4269/ajtmh.13-0070 24062479 PMC3820334

[B60] Oliveira E. Oliveira D. Cardoso F. A. Barbosa J. R. Marcelino A. P. Dutra T.. (2017). Multicentre evaluation of a direct agglutination test prototype kit (DAT-LPC) for diagnosis of visceral leishmaniasis. Parasitology 144, 1964–1970. 10.1017/S0031182017001378 28735574

[B61] Oliveira E. Pedras M. J. Assis I. E. M. Rabello A. (2009). Improvement of direct agglutination test (DAT) for laboratory diagnosis of visceral leishmaniasis in Brazil. Trans. R. Soc. Trop. Med. Hyg. 103, 1279–1281. 10.1016/j.trstmh.2009.04.007 19457530

[B62] Oliveira E. Saliba S. W. Andrade C. F. Rabello A. (2011). Direct agglutination test (DAT): improvement of biosafety for laboratory diagnosis of visceral leishmaniasis. Trans. R. Soc. Trop. Med. Hyg. 105, 414–416. 10.1016/j.trstmh.2011.04.010 21616516

[B63] Oliveira E. Saliba S. W. Saliba J. W. Rabello A. (2013). Validation of a direct agglutination test prototype kit for the diagnosis of visceral leishmaniasis. Trans. R. Soc. Trop. Med. Hyg. 107, 243–247. 10.1093/trstmh/trt004 23382276

[B64] Pedras M. J. de Gouvêa Viana L. de Oliveira E. J. Rabello A. (2008). Comparative evaluation of direct agglutination test, rK39 and soluble antigen ELISA and IFAT for the diagnosis of visceral leishmaniasis. Trans. R. Soc. Trop. Med. Hyg. 102, 172–178. 10.1016/j.trstmh.2007.11.004 18160087

[B65] Peruhype-Magalhães V. Machado-de-Assis T. S. Rabello A. (2012). Use of the kala-azar detect® and IT-LEISH® rapid tests for the diagnosis of visceral leishmaniasis in Brazil. Mem. Inst. Oswaldo Cruz 107, 951–952. 10.1590/S0074-02762012000700019 23147155

[B66] Pinedo-Cancino V. Kesper N. Barbiéri C. L. Lindoso J. A. L. Umezawa E. S. (2013). The efficacy of L. *(L.)* chagasi excreted-secreted antigens (ESAs) for visceral leishmaniasis diagnosis is due to low levels of cross-reactivity. Am. J. Trop. Med. Hyg. 88, 559–565. 10.4269/ajtmh.12-0587 23324219 PMC3592541

[B67] Pomares C. Despierres L. del Giudice P. Delaunay P. Michel G. Ferrua B.. (2012). Western blot analysis as an aid for the diagnosis of cutaneous leishmaniasis due to *Leishmania major*. Trans. R. Soc. Trop. Med. Hyg. 106, 452–454. 10.1016/j.trstmh.2012.03.001 22657532

[B68] Portela Á. S. B. Costa L. E. Salles B. C. S. Lima M. P. Santos T. T. O. Ramos F. F.. (2018). Identification of immune biomarkers related to disease progression and treatment efficacy in human visceral leishmaniasis. Immunobiology 223, 303–309. 10.1016/j.imbio.2017.10.043 29074301

[B69] Ribeiro P. A. F. Dias D. S. Lage D. P. Costa L. E. Salles B. C. S. Steiner B. T.. (2018). A conserved leishmania hypothetical protein evaluated for the serodiagnosis of canine and human visceral and tegumentary leishmaniasis, as well as a serological marker for the posttreatment patient follow-up. Diagn. Microbiol. Infect. Dis. 92, 196–203. 10.1016/j.diagmicrobio.2018.05.026 29941364

[B70] Riera C. Fisa R. López-Chejade P. Serra T. Girona E. Jiménez M.. (2008). Asymptomatic infection by *Leishmania infantum* in blood donors from the Balearic Islands (Spain). Transfusion 48, 1383–1389. 10.1111/j.1537-2995.2008.01708.x 18422844

[B71] Saghrouni F. Khammari I. Kaabia N. Bouguila J. Ben Abdeljelil J. Fathallah A.. (2012). Asymptomatic carriage of *Leishmania* in family members of patients with visceral leishmaniasis in Central Tunisia. Pathol. Biol. 60, e55–58. 10.1016/j.patbio.2011.11.001 22154335

[B72] Sakkas H. Gartzonika C. Levidiotou S. (2016). Laboratory diagnosis of human visceral leishmaniasis. J. Vect. Borne Dis. 53, 8–16. 27004573

[B73] Sakru N. Korkmaz M. Ozbel Y. Ertabaklar H. Sengul M. Toz S. O. (2007). Investigation of asymptomatic visceral leishmaniasis cases using western blot in an endemic area in Turkey. New Microbiol. 30, 13–18. 17319595

[B74] Santos-Gomes G. Gomes-Pereira S. Campino L. Araújo M. D. Abranches P. (2000). Performance of immunoblotting in diagnosis of visceral Leishmaniasis in human immunodeficiency virus-*Leishmania* sp.-coinfected patients. J. Clin. Microbiol. 38, 175–178. 10618083 10.1128/jcm.38.1.175-178.2000 PMC86049

[B75] Sarkari B. Ashrafmansouri M. Hatam G. Habibi P. Abdolahi Khabisi S. (2014). Performance of an ELISA and indirect immunofluorescence assay in serological diagnosis of zoonotic cutaneous leishmaniasis in Iran. Interdiscip. Perspect. Infect. Dis. 2014:505134. 10.1155/2014/505134 25177349 PMC4142716

[B76] Sarkari B. Rezaei Z. Mohebali M. (2018). Immunodiagnosis of visceral leishmaniasis: current status and challenges: a review article. Iran J. Parasitol. 13, 331–341. 30483323 PMC6243177

[B77] Sato C. M. Sanchez M. C. A. Celeste B. J. Duthie M. S. Guderian J. Reed S. G.. (2017). Use of recombinant antigens for sensitive serodiagnosis of american tegumentary leishmaniasis caused by different *Leishmania* species. J. Clin. Microbiol. 55, 495–503. 10.1128/JCM.01904-16 27927927 PMC5277519

[B78] Seyyedtabaei S. J. Rostami A. Haghighi A. Mohebali M. Kazemi B. Fallahi S.. (2017). Detection of potentially diagnostic *Leishmania* Antigens with western blot analysis of sera from patients with cutaneous and visceral leishmaniases. Iran J. Parasitol. 12, 206–214. 28761480 PMC5527030

[B79] Silva E. S. Schoone G. J. Gontijo C. M. Brazil R. P. Pacheco R. S. Schallig H. D. (2005). Application of Direct Agglutination Test (DAT) and Fast Agglutination Screening Test (FAST) for sero-diagnosis of visceral leishmaniasis in endemic area of Minas Gerais, Brazil. Kinetoplas. Biol. Dis. 4:4. 10.1186/1475-9292-4-4 15955248 PMC1183242

[B80] Singh O. P. Hasker E. Sacks D. Boelaert M. Sundar S. (2014). Asymptomatic *Leishmania* infection: a new challenge for *Leishmania* control. Clin. Infect. Dis. 58, 1424–1429. 10.1093/cid/ciu102 24585564 PMC4001287

[B81] Stensvold C. R. Høst A. V. Belkessa S. Nielsen H. V. (2019). Evaluation of the NovaLisa^TM^ *Leishmania Infantum* IgG ELISA in a reference diagnostic laboratory in a non-endemic country. Antibodies 8:20. 10.3390/antib8010020 31544826 PMC6640698

[B82] Tebourski F. el Gaied A. Louzir H. Ben Ismail R. Kammoun R. Dellagi K. (1994). Identification of an immunodominant 32-kilodalton membrane protein of *Leishmania donovani infantum* promastigotes suitable for specific diagnosis of Mediterranean visceral leishmaniasis. J. Clin. Microbiol. 32, 2474–2480. 10.1128/JCM.32.10.2474-2480.1994 7814485 PMC264086

[B83] Varani S. Ortalli M. Attard L. Vanino E. Gaibani P. Vocale C.. (2017). Serological and molecular tools to diagnose visceral leishmaniasis: 2-years' experience of a single center in Northern Italy. PLoS ONE 12:e0183699. 10.1371/journal.pone.0183699 28832646 PMC5568375

[B84] World Health Organization (2010b). Visceral Leishmaniasis Rapid Diagnostic Test Performance WHO Diagnostic Evaluation Series.

[B85] World Health Organization (Ed.) (2010a). Control of the Leishmaniases: Report of a Meeting of the WHO Expert Committee on the Control of Leishmaniases, Geneva, 22 - 26 March 2010. Geneva: World Health Organization.

[B86] Zanetti A. D. S. Sato C. M. Longhi F. G. Ferreira S. M. B. Espinosa O. A. (2019). Diagnostic accuracy of enzyme-linked immunosorbent assays to detect anti-*Leishmania* antibodies in patients with American tegumentary leishmaniasis: a systematic review. Rev. Inst. Med. Trop. São Paulo 61:e42. 10.1590/s1678-9946201961042 31432991 PMC6710007

